# Levels of peripheral blood routine, biochemical and coagulation parameters in patients with hemorrhagic fever with renal syndrome and their relationship with prognosis: an observational cohort study

**DOI:** 10.1186/s12879-023-08777-w

**Published:** 2024-01-11

**Authors:** Wen-jing Chen, Hong Du, Hai-feng Hu, Jian-qi Lian, Hong Jiang, Jing Li, Yan-ping Chen, Ying Zhang, Ping-zhong Wang

**Affiliations:** 1 Center for Infectious Diseases, the Second Affiliated Hospital of Air Force Medical University, 569 Xinsi Rd, Baqiao District, Xi’an, 710038 Shaanxi China; 2 Department of Infectious Diseases, the Second Affiliated People’s Hospital of Yan ‘an University, Yan’an, Shaanxi China

**Keywords:** Hemorrhagic fever with renal syndrome, Coagulation parameters, Hyperfibrinolysis, Risk factors, Prognosis

## Abstract

**Background:**

Hantaan virus (HTNV), Seoul virus (SEOV) and Puumala virus (PUUV) are major serotypes of the Hantavirus, which can cause hemorrhagic fever with renal syndrome (HFRS). The pathophysiology of HFRS in humans is complex and the determinants associated with mortality, especially the coagulation and fibrinolysis disorders, are still not been fully elucidated. Severe patients usually manifest multiple complications except for acute kidney injury (AKI). The aim of this study was to observe the levels of peripheral blood routine, biochemical and coagulation parameters during the early stage, so as to find independent risk factors closely related to the prognosis, which may provide theoretical basis for targeted treatment and evaluation.

**Methods:**

A total of 395 HFRS patients from December 2015 to December 2018 were retrospectively enrolled. According to prognosis, they were divided into a survival group (*n* = 368) and a death group (*n* = 27). The peripheral blood routine, biochemical and coagulation parameters were compared between the two groups on admission. The relationship between the parameters mentioned above and prognosis was analyzed, and the dynamic changes of the coagulation and fibrinolysis parameters during the first week after admission were further observed.

**Results:**

In addition to AKI, liver injury was also common among the enrolled patients. Patients in the death group manifested higher levels of white blood cell counts (WBC) on admission. 27.30% (107/392) of the patients enrolled presented with disseminated intravascular coagulation (DIC) on admission and DIC is more common in the death group; The death patients manifested longer prothrombin time (PT) and activated partial thromboplastin time (APTT), higher D-dimer and fibrinogen degradation product (FDP), and lower levels of platelets (PLT) and fibrinogen (Fib) compared with those of the survival patients. The proportion of D-dimer and FDP abnormalities are higher than PT, APTT and Fib. Prolonged PT, low level of Fib and elevated total bilirubin (TBIL) on admission were considered as independent risk factors for prognosis (death).

**Conclusions:**

Detection of PT, Fib and TBIL on admission is necessary, which might be benefit to early predicting prognosis. It is also important to pay attention to the dynamic coagulation disorders and hyperfibrinolysis during the early stage in the severe HFRS patients.

## Background

Hantavirus can cause two diseases in humans: Hemorrhagic fever with renal syndrome (HFRS) and Hantavirus Cardiopulmonary Syndrome (HCPS) [1]. HCPS is mainly caused by Sin Nombre virus (SNV), which is prevalent in the Americas, with a high mortality rate of more than 30%-50% [1]; HFRS is mainly caused by Hantaan virus (HTNV), Seoul virus (SEOV), Puumala virus (PUUV) and other viral serotypes of the Hantavirus [2]. HFRS caused by PUUV infection usually has mild manifestation and is mainly prevalent in Europe, also known as epidemic nephropathy (NE), with a fatality rate of about 0.1% [3]. HFRS in China is mainly caused by HTNV and SEOV infection, and the patients infected by HTNV are more serious and the fatality rate can reach 5%-10% [4].

The pathogenesis of HFRS is complex and has not been fully elucidated. Currently, it has been confirmed that hantavirus infection can cause increased vascular endothelial permeability, inflammatory cytokine storm, severe thrombocytopenia and coagulation disorder [4, 5]. The refractory shock, hemorrhage related complications, disseminated intravascular coagulation (DIC) and multiple organ dysfunction syndrome (MODS) have been considered as major causes of death in the severe HFRS patients [4, 6].

There is still no specific treatment for HFRS. Early detection, early diagnosis and early symptomatic support treatment are still the main treatment principles. The pathophysiology of HFRS in humans is complex and the determinants associated with mortality, especially the coagulation and fibrinolysis disorders, are still not been fully elucidated. Based upon the background, we aimed to observe the levels of peripheral blood routine, biochemical and coagulation parameters during the early stage, so as to find independent risk factors closely related to the prognosis. Furthermore, the dynamic changes of coagulation and fibrinolysis parameters were observed and analyzed, which will also be benefit to providing theoretical basis for targeted evaluation and intervention.

## Methods

### Study design

Clinical data of the adult patients diagnosed with HFRS, from December 2015 to December 2018 were retrospectively collected. All the patients were treated in the Center for Infectious Diseases of the Second Affiliated Hospital of Air Force Medical University. Diagnosis was based upon epidemiological history, clinical manifestations and laboratory examination; The patients enrolled were positive for both Hantavirus-specific IgM and IgG by colloidal gold assay on admission and a total of 406 cases were eligible initially.

The patients who were pregnant, under 18 years old, used anticoagulants before hospitalization, combined with other viral diseases, hematological diseases and neoplastic diseases were excluded. Finally, 395 HFRS patients were enrolled excluding six cases under 18 years old, three pregnant cases and two cases combined with liver cancer.

We only observed the mortality of patients during hospitalization without follow-up. Most of the patients were discharged after their condition improved. The patients diagnosed with severe-type and critical-type will be treated in the intensive care unit (ICU), who will transfer to the general ward when their clinical conditions were stabilized.

These patients enrolled were divided into a survival group (*n* = 368) and a death group (*n* = 27) according to the prognosis (death). The levels of main peripheral blood routine, biochemical and coagulation parameters were tested and compared between the two groups on admission, and there laboratory indicators were also compared with normal reference ranges. The relationship between the above-mentioned parameters and prognosis (death) was also analyzed.

The dynamic changes of coagulation and fibrinolysis indexes during the first week after admission were further observed aiming at 272 HFRS patients with completed record on detection of hematological parameters, and DIC was defined by the DIC scoring system of the International Society of Thrombosis and Hemostasis (ISTH) [7].

### Blood analysis

The above indicators were tested in the central laboratory of the hospital, and professional inspectors operated them accordance with the instructions. 3 mL venous blood was collected from the patient on admission. Hantavirus-specific IgM and IgG were tested by colloidal gold assay (Kit purchased from Xiamen Bosheng Biotechnology Co., LTD.) The PCT was detected by VIDAS automatic enzyme-linked fluorescence analyzer produced by Merieux Biologic in France, and CRP was detected by Beckman Coulter Immage800 special protein analyzer in the United States. The patients' liver and kidney function, blood routine and coagulation indexes were simultaneously detected by automatic biochemical analyzer (Cimekang, XT-4000i, Japan; Hitachi, 7600–100, Japan) and blood analyzer testing (Cismecon, CA7000, Japan; ACL, TOP700, USA).

### Data collection

Most patients with HFRS are admitted to our department after being diagnosed in the emergency department or infection disease clinic, a small number of the patients were transferred to our department after being diagnosed in other departments or from other hospitals. Clinical information and parameters were extracted from physician documentation via reviewing from the electronic medical record by study investigators.

### Statistical analysis

SPSS 23.0 was used for statistical analysis, and GraphPad Prism 9.0 was used for graphing. Categorical variables were expressed as percentages, and Fisher's test, continuity correction or Pearson's chi-square test were used for intergroup analysis. Continuous variables were tested for normal distribution and homogeneity of variance, and those with normal distribution were expressed as mean ± standard deviation, and the Student's t test was used for the comparison between the two groups; the variables with non-normal distribution were expressed as median and interquartile range, and the Mann–Whitney U test was used for the comparison between the two groups.

Logistic regression was used to analyze the independent risk factors for prognosis (death). Considering the possible influence of multicollinearity on regression analysis and subsequent results, variance inflation factor (VIF) was used to detect multicollinearity between variables, and the variables that were statistically significant (*P* < 0.1) related to death in the univariate analysis without multicollinearity were further included in the multivariate logistic regression analysis. Relaxing the *P*-value would allow for as many alternative confounding factors as possible.

In the logistic regression analysis model, the regression coefficient was estimated by the maximum likelihood method. When the number of the independent variables was large, the stepwise regression analysis was used to select risk factors. Logistic regression model was tested using the likelihood ratio test.

The receiver operating characteristic (ROC) curve was used to detect the predictive power of the variables on prognosis, and the area under the ROC curve (AUC) and 95% confidence interval (CI) were calculated. The hypothesis test was two-sided, and *P* < 0.05 was considered statistically significant.

## Results

### Demographic and prognostic analysis of the HFRS patients

The length of stay from admission to discharge was 11 days (IQR 9–15) in the survival group and 3 days (IQR 1–8) in the death group. The mean age of the patients in the survival group and the death group was 44 years vs. 45 years, with no significant difference (*P* = 0.812). The mean duration from illness onset to the day of admission in the survival group and the death group was 5.83 days vs. 6.19 days, with no significant difference (*P* = 0.415). During the study period, 27 patients died, with total fatality rate of 6.84% (27/395) (Table 1).

**Table 1:** Laboratory results and abnormalities on admission in the HFRS patients

Variables	Survived	Died	Total	*P* value
Sex				
Male, n (%)	295/368 (80.16)	21/27 (77.78)	316/395 (80.00)	0.765^†^
Female, n (%)	73/368 (19.84)	6/27 (22.22)	79/395 (20.00)	
Age (years)				
Mean (SD)	43.80 (15.05)	44.52 (16.54)	43.85 (15.14)	0.812^*^
Length of stay (days)				
Median (IQR)	11 (9–15)	3 (1–8)	11 (8–15)	< 0.001^‡^
Timing (days)^a^				
Mean (SD)	5.83 (1.62)	6.19 (3.19)	5.61 (1.72)	0.415^*^
WBC				
Median, × 10^9^/L (IQR)	13.29 (8.79–19.48)	23.19 (10.99–36.72)	13.62 (8.81–20.67)	0.002^‡^
≤ 10, n (%)	117/368 (31.79)	7/27 (25.93)	124/395 (31.39)	< 0.001^†^
> 10 to ≤ 30, n (%)	213/368 (57.88)	10/27 (37.04)	223/395 (56.46)	
> 30, n (%)	38/368 (10.33)	10/27 (37.04)	48/395 (12.15)	
HCT				
Mean. % (SD)	40.39 (6.68)	41.49 (10.18)	40.46 (6.96)	0.590^*^
PLT				
Median. × 10^9^/L (IQR)	41 (22–70)	22 (15–30)	39 (21–68)	0.003^‡^
≥ 50,n (%)	150/368 (40.76)	7/27 (25.93)	157/395 (39.75)	0.154^†^
≥ 20 to < 50, n (%)	136/368 (36.96)	10/27 (37.04)	146/395 (36.96)	
< 20, n (%)	82/368 (22.28)	10/27 (37.04)	92/395 (23.29)	
ALB				
Mean. g/L (SD)	29.97 (6.02)	28.13 (7.46)	29.85 (6.13)	0.139^*^
< 30 g/L, n (%)	193/368 (52.45)	18/27 (66.67)	211/395 (53.42)	0.153^†^
≥ 30 g/L, n (%)	175/368 (47.55)	9/27 (33.33)	184/395 (46.58)	
ALT				
Median. IU/L (IQR)	56 (41–83)	76 (49–379)	57 (41–86)	0.017^‡^
≤ 40, n (%)	84/366 (22.95)	5/26 (19.23)	89/392 (22.70)	0.662^†^
> 40, n (%)	282/366 (77.05)	21/26 (80.77)	303/392 (77.30)	
AST				
Median. IU/L (IQR)	79 (56–124)	167 (84–911)	80 (56–135)	0.001^‡^
≤ 40, n (%)	45/366 (12.30)	1/26 (3.85)	46/392 (11.73)	0.328^†^
> 40, n (%)	321/366 (87.70)	25/26 (96.15)	346/392 (88.27)	
TBIL				
Median. μmmol/L (IQR)	12.75 (9.63–16.67)	15.10 (11.80–28.53)	12.85 (9.80–17.10)	0.006^‡^
≤ 17.1, n (%)	280/366 (76.50)	13/25 (52.00)	293/391 (74.94)	0.009^†^
> 17.1, n (%)	86/366 (23.50)	12/25 (48.00)	98/391 (25.06)	
Potassium				
Median. mmol/L (IQR)	3.65 (3.34–4.01)	3.42 (3.13–3.95)	3.64 (3.33–4.00)	0.240^‡^
> 5.5, n (%)	3/362 (0.83)	2/27 (7.41)	5/389 (1.29)	0.001†
< 3.5, n (%)	135/362 (37.29)	15/27 (55.56)	150/389 (38.56)	
Sodium				
Mean. mmol/L (SD)	133.12 (6.30)	132.04 (6.69)	133.0 5 (6.33)	0.393^*^
< 135, n (%)	211/362 (58.29)	19/27 (70.37)	240/389 (61.70)	0.337^†^
SCr				
Median. μmol/L (IQR)	160.50 (91.30–325.20)	222.00 (91.80–309.40)	166.65 (91.08–325.05)	0.779^‡^
RIFLE-1, n (%)	34/367 (9.26)	0/27 (0.00)	34/394 (8.63)	0.216^†^
RIFLE-2, n (%)	65/367 (17.71)	8/27 (29.63)	73/394 (18.53)	
RIFLE-3, n (%)	87/367 (23.71)	6/27 (22.22)	93/394 (23.60)	
PT				
Median. sec (IQR)	11.60 (10.90–12.90)	13.80 (12.70–20.50)	11.70 (10.90–13.00)	< 0.001^‡^
≤ 13, n (%)	279/365 (76.44)	7/26 (26.92)	286/391 (73.15)	< 0.001^†^
> 13 to ≤ 19, n (%)	84/365 (23.01)	12/26 (46.15)	96/391 (24.55)	
> 19, n (%)	2/365 (0.55)	7/26 (26.93)	9/391 (2.30)	
APTT				
Median. sec (IQR)	37.90 (30.60–51.60)	54.30 (38.30–80.50)	38.80 (30.60–53.40)	0.003^‡^
≤ 37, n (%)	174/365 (47.67)	6/26 (23.08)	180/391 (46.04)	0.015^†^
> 37, n (%)	191/365 (52.33)	20/26 (76.92)	211/391 (53.96)	
TT				
Median. sec (IQR)	21.55 (17.60–28.25)	35.70 (22.90–68.50)	21.70 (17.88–30.43)	< 0.001^‡^
Fib				
Mean. g/L (SD)	2.63 (1.02)	1.69 (0.65)	2.57 (1.02)	0.001^*^
≥ 2, n (%)	265/363 (73.00)	12/26 (46.15)	277/389 (71.21)	< 0.001^†^
≥ 1 to < 2, n (%)	88/363 (24.24)	8/26 (30.77)	96/389 (24.68)	
< 1, n (%)	10/363 (2.75)	6/26 (23.08)	16/389 (4.11)	
D-dimer				
Median. ug/ml (IQR)	3.67 (2.34–6.10)	5.72 (2.93–13.30)	3.76 (2.39–6.26)	0.093^‡^
≤ 0.5, n (%)	4/363 (1.10)	0/26 (0.00)	4/389 (1.03)	0.042^†^
> 0.5 to ≤ 5, n (%)	235/363 (64.74)	11/26 (42.31)	246/389 (63.24)	
> 5 to ≤ 9, n (%)	73/363 (20.11)	6/26 (23.08)	79/389 (20.31)	
> 9, n (%)	51/363 (14.05)	9/26 (34.62)	60/389 (15.42)	
FDP				
Median. ug/ml (IQR)	10.50 (6.98–15.68)	13.09 (7.60–30.00)	10.71 (7.00–16.08)	0.080^‡^
≤ 5, n (%)	42/363 (11.57)	1/26 (3.85)	43/389 (11.05)	0.374^†^
> 5, n (%)	321/363 (88.43)	25/26 (96.15)	346/389 (88.95)	
CRP				
Median. mg/L (IQR)	26.17 (13.90–41.32)	30.89 (11.22–59.98)	26.17 (13.62–40.04)	0.591^‡^
DIC				
Yes, n (%)	91/367 (24.80)	16/27 (59.26)	107/392 (27.30)	< 0.001^†^

RIFLE-1 acute kidney injury 1.5–2 × baseline creatinine, RIFLE-2 acute kidney injury 2–3 × baseline creatinine, RIFLE-3 acute kidney injury > 3 × baseline creatinine

*WBC* White blood cells, *HCT* Haematocrit, *PLT* Platelets, *ALT* Alanine transaminase, *AST* Aspartate transaminase, *ALB* Albumin, *TBIL* Total bilirubin, *SCr* Serum Creatinine, *PT* Prothrombin time, *APTT* Activated partial thromboplastin time, *TT* Thrombin time, *Fib* Fibrinogen, *FDP* Fibrinogen degradation products, *CRP* C-reactive protein

^a^Timing: Duration from illness onset to the day of admission

^*^Denotes *p* value from student's *t* test

^†^Denotes *p* value from χ^2^ test

^‡^Deontes *p* value from Mann–Whitney U test

### Analysis of blood routine, biochemical and coagulation parameters on admission in HFRS patients

**Fig. 1:**
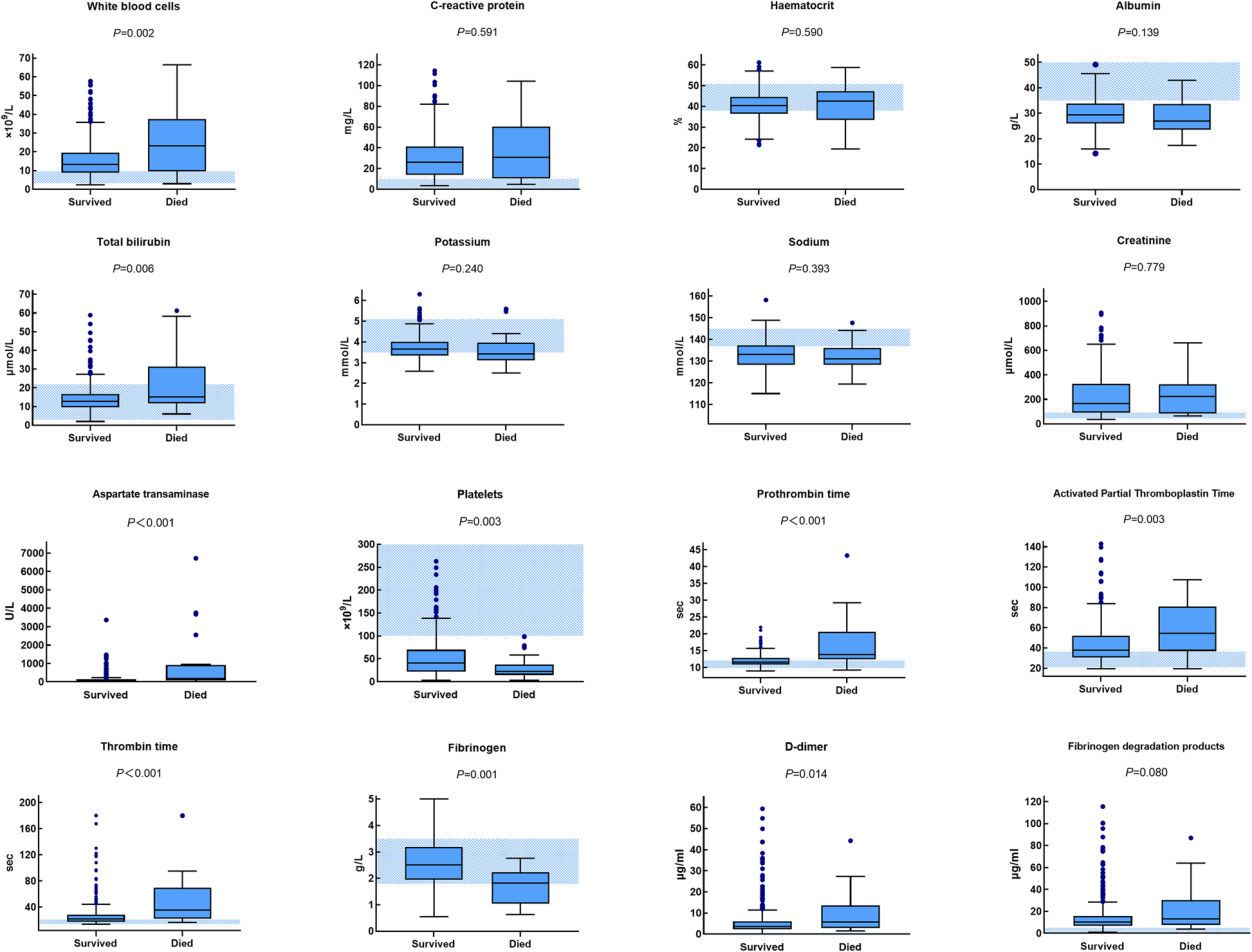
Box plots of peripheral blood routine, biochemical and coagulation parameters on admission in the HFRS patients

AKI was more common in the HFRS patients (200/394[50.76%]), the median level of SCr was 160.50 mmol/L vs. 220.00 mmol/L in the survival group and the death group, respectively (*P* = 0.779). Severe AKI (RIFLE 3 stage) occurred in 22.22% (6/27) of the patients in the death group and 23.71% (87/367) of the patients in the survival group. 39.85% (155/389) of the patients had abnormal serum potassium on admission, and the incidence of hypokalemia was higher than that of hyperkalemia. 61.70% (240/389) of the patients had hyponatremia on admission (Fig. 1, Table 1).

Abnormal liver function was also common, with 77.30% (303/392) of the patients manifesting abnormal ALT on admission. The median level of ALT in the survival group and the death group was 56U/L vs. 76U/L (*P* = 0.017), and 88.27% (346/392) of the patients had abnormal AST on admission, with median levels of 79U/L vs. 167U/L in the survival group and the death group, respectively (*P* = 0.001). Median level of TBIL and mean concentration of ALB were 12.75μmmol/L vs. 15.10μmmol/L (*P* = 0.006), and 29.97 g/L vs. 28.13 g/L (*P* = 0.139) between the survival group and the death group, respectively. 25.06% (98/391) of the patients had elevated level of TBIL on admission. (Fig. 1, Table 1).

The mean level of WBC on admission was 13.29 × 10^9^/L vs. 23.19 × 10^9^/L in the survival group and the death group, respectively (Fig. 1, Table 1). 68.61% (271/395) of the patients had abnormal expression of WBC, and 37.04% (10/27) of the dead group had level of WBC above 30 × 109/L, but only 10.33% (38/368) of the survival group. Mean level of HCT in the survival and the death group was 40.39% vs. 40.49% (*P* = 0.590), and median level of CRP in the survival and the death group was 26.17 mg/L vs. 30.89 mg/L (*P* = 0.591) respectively (Fig. 1, Table 1).

60.25% (238/395) of the HFRS patients had low level of PLT(< 50 × 109/L), the median time of PT, APTT and TT were 11.60 s vs. 13.80 s (*P* < 0.001), 37.90 s vs. 54.30 s (*P* < 0.001) and 21.55 s vs. 35.70 s (*P* < 0.001) in the death group and the survival group, respectively. 53.96% (211/391) of the patients had abnormal APTT; 26.85% (105/391) of the patients had abnormal PT. 28.79% (112/389) of the patients had low level of Fib (< 2 g/L) on admission, with mean level of 2.63 g/L vs. 1.69 g/L in the survival group and the death group, respectively (*P* < 0.001). The median level of FDP and D-dimer were 10.50/ml vs. 13.09 μg/ml (*P* = 0.080), and 3.67 μg/ml vs. 5.72 μg/ml in the survival group and the death group, respectively (*P* = 0.093). 98.97% (385/389) of the patients had abnormal D-dimer and 88.95% (346/389) of the patients had abnormal FDP (Fig. 1, Table 1). 27.30% (107/392) of the patients enrolled had DIC on admission, including 59.26% (16/27) of the death group and 24.80% (91/367) of the survival group (Table 1).

Boxes represent median, 25th, and 75th centiles. Whiskers represent maximum values excluding outliers, which are represented by dots. Outliers are defined as greater than 75th centile plus 1.5 times the interquartile range. Shaded area indicates normal range for the China population. P values are from t tests (potassium, sodium, haematocrit, fibrinogen) or the Mann–Whitney U test (all the others).

### Univariate and multivariate logistic regression analysis of prognostic risk factors in the HFRS patients

Univariate analysis showed that increased WBC, decreased PLT, decreased Fib, increased AST, increased TBIL, prolonged PT, prolonged APTT and prolonged TT were correlated with clinical prognosis (death) (Table 2).

**Table 2:** Univariate and multivariate logistic regression analysis for prognostic risk factors in the HFRS patients

Variables	Univariate analysis		Multivariate analysis	
	OR (95% CI)	*P* value	OR (95% CI)	*P* value
Female	1.155 (0.450–2.964)	0.765		
Age	1.003 (0.978–1.029)	0.811		
WBC	1.047 (1.023–1.072)	< 0.001		
PLT	0.981 (0.965–0.997)	0.023		
ALT	1.004 (1.001–1.006)	0.002		
AST	1.001 (1.001–1.002)	< 0.001		
TBIL	1.064 (1.032–1.098)	< 0.001	1.067 (0.996–1.143)	0.063
PT	1.424 (1.246–1.626)	< 0.001	1.317 (1.076–1.613)	0.008
APTT	1.017 (1.006–1.029)	0.003		
TT	1.010 (1.004–1.017)	0.002		
Fib	0.266 (0.148–0.478)	< 0.001	0.246 (0.085–0.711)	0.010
FDP	1.010 (0.999–1.022)	0.063		
CRP	1.014 (0.999–1.029)	0.067		

*OR* odds ratio, The OR value reflects how high the risk of an outcome occurring is with exposure versus without exposure. *CI* confidence interval; *P* values are from likelihood ratio tests

*WBC* White blood cells, *PLT* Platelets, *AST* Aspartate transaminase, *ALB* Albumin, *TBIL* Total bilirubin, *PT* Prothrombin time, *APTT* Activated partial thromboplastin time, *TT* Thrombin time, *Fib* Fibrinogen, *FDP* Fibrinogen degradation products, *CRP* C-reactive protein

Multicollinearity test showed that the multicollinearity was not significant (VIF values for all the variance inflation factors were below 2.253). Finally, the increased TBIL, prolonged PT and decreased Fib were screened out to be independent risk factors for death in the HFRS patients by multivariate analysis (Table 2).

### Predictive efficacy of PT, Fib and TBIL for prognosis by ROC analysis

**Fig. 2:**
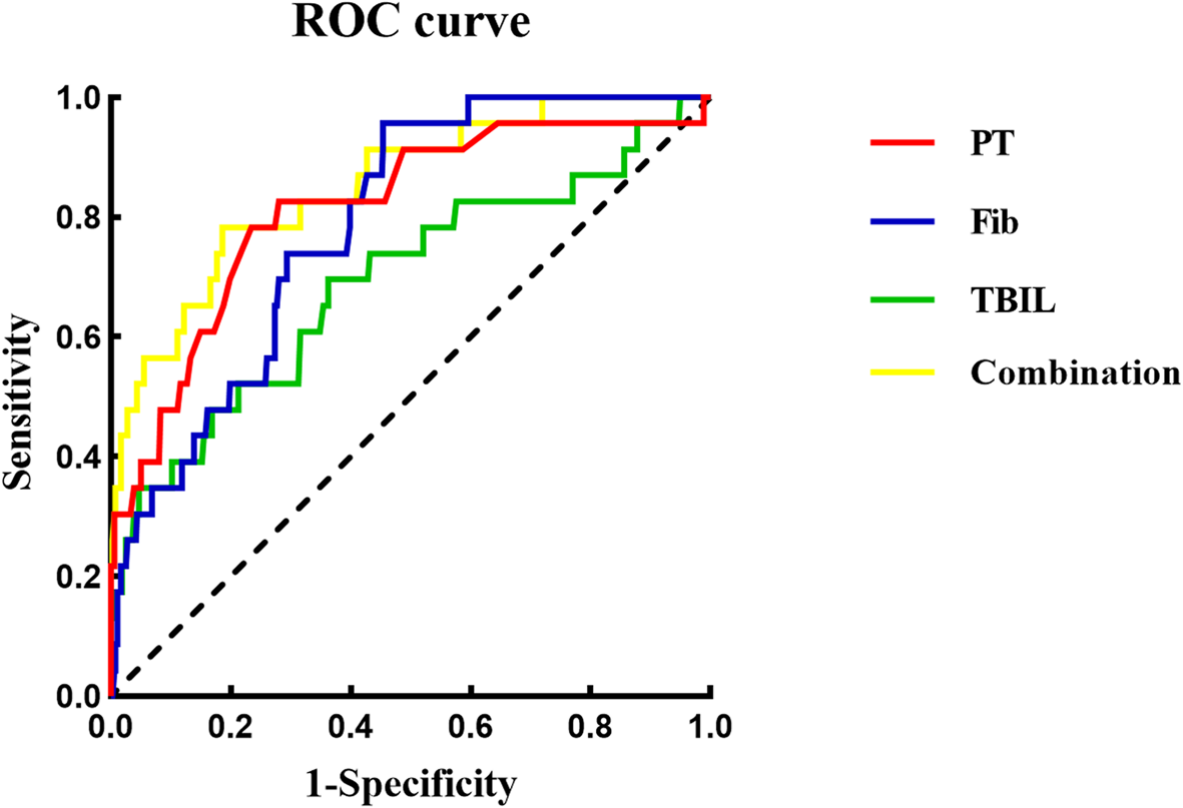
Predictive efficacy of PT, Fib, TBIL and their combination on admission for prognosis (death) in HFRS patients by ROC analysis

ROC analysis showed that the AUC values of PT, Fib and TBIL for predicting prognosis (death) were 0.814 (95% CI 0.714–0.914, *P* < 0.001), 0.787 (95% CI 0.710–0.863, *P* < 0.010) and 0.692 (95% CI 0.564–0.819, *P* = 0.002), respectively. The predictive AUC value was 0.852 (95% CI 0.768–0.937, *P* < 0.001) by the three parameters in combination, with sensitivity of 0.783 and specificity of 0.937 (Table 3, Fig. 2).

**Table 3:** Predictive efficacy of PT, Fib and TBIL for prognosis (death) by ROC analysis

Variables	AUC	*P* value	Cut-off value	Sensitivity	Specificity	95% CI for AUC	
						Lower	Upper
PT	0.814	< 0.001	12.950	0.783	0.765	0.714	0.914
Fib	0.787	< 0.001	2.428	0.957	0.546	0.710	0.863
TBIL	0.692	0.002	14.655	0.696	0.637	0.564	0.819
Combination^a^	0.853	< 0.001	0.030^b^	0.783	0.814	0.768	0.937

*AUC* area under the receiver operating characteristic, *CI* confidence interval, *TBIL* Total bilirubin, *PT* Prothrombin time, *Fib* Fibrinogen

^a^PT, Fib and TBIL in combination

^b^Probability value of the combination was analyzed by logistic regression

### Dynamic changes of peripheral coagulation parameters in HFRS patients during the first week after admission

The dynamic changes of the coagulation parameters were observed in 272 HFRS patients in this study, and all the 14 patients died within the first week after admission; Considering the rapid progression of the disease within one week of admission, we investigated the dynamics of coagulation parameters within one week of admission in HFRS patients.

**Fig. 3:**
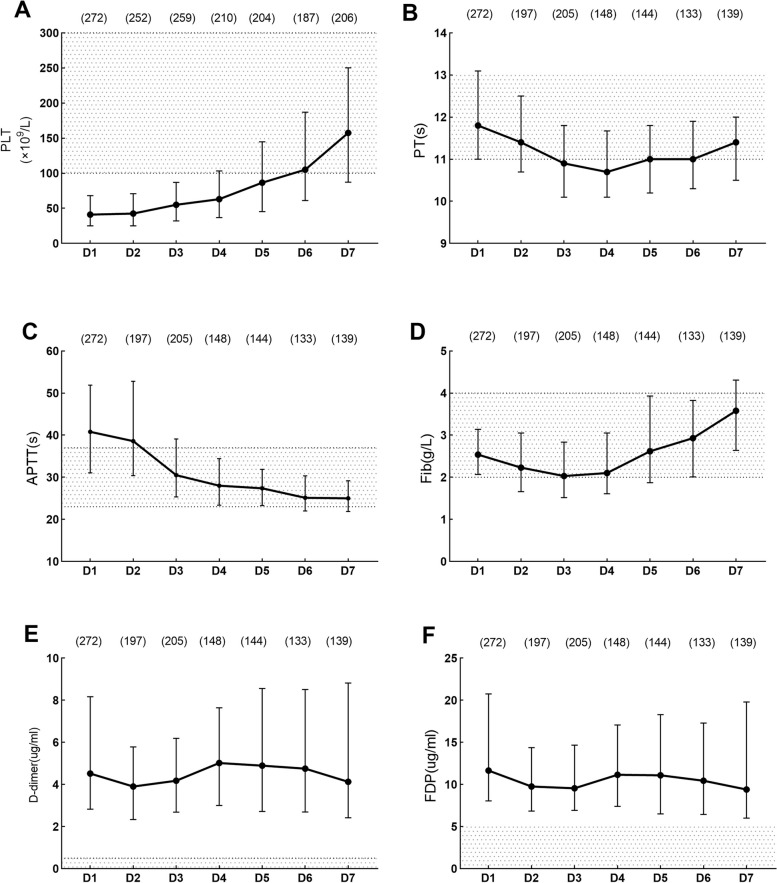
Dynamic changes of peripheral coagulation parameters in the HFRS patients during the first week after admission

PLT decreased to the lowest value on the 1st to 2nd day after admission, and began to increase on the 4th day. PT and APTT increased to the highest value on the first day after admission. Fib decreased to the lowest value on the third day after admission. PLT, PT, APTT and Fib tended to be normal value on the 7th day after admission. D-dimer and FDP increased from the first day after admission, followed by slight fluctuation, and then remained to a high level within the first week after admission (Fig. 3).

The error bars show medians and 25% and 75% percentiles. The number of the patients sampled from D1 to D7 is shown on the top of the graph. The shaded area indicates normal range for the coagulation parameters.

The median length of stay in the non-survival patients after admission was 3 days. Since the relatively few death cases enrolled and significant lacking of data after the 5th day of admission, so the dynamic change of coagulation parameters was only drawn within the 5th day after admission.

**Fig. 4:**
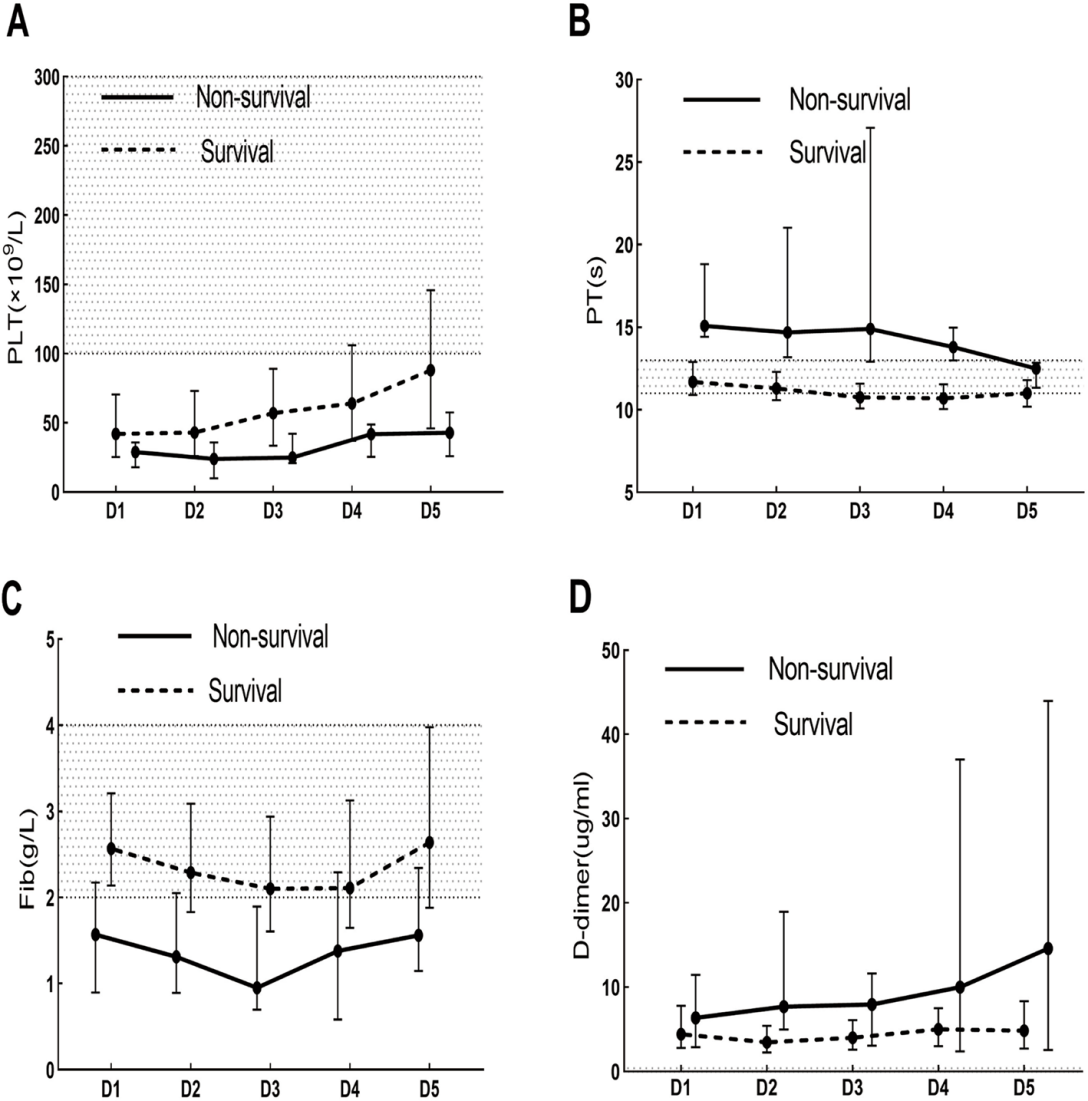
Dynamic changes of the peripheral coagulation parameters in the survivors and non-survivors from day 1 to day 5 after admission

The death patients manifested longer PT, higher level of D-dimer, and lower levels of PLT and Fib within the 5th days after admission compared with those of the survival patients (Fig. 4). Because of lacking results of APTT and FDP in most of the non-survival patients, the corresponding line graph was not drawn in the figure.

The shaded area indicates normal range for the coagulation parameters.

## Discussion

In this study, we explored the predictors for prognosis based on the routine blood chemistry and hematological parameters from patients infected with HTNV on admission and the dynamic changes of the coagulation and fibrinolysis parameters within 1 week after admission.

The kidney is the major organ involved in HFRS. This study showed that there was no significant difference in the level of SCr between the survival group and the death group. Some studies even showed that the level of SCr in the dead patients was lower than that of the survival patients [8]. The reason may be that in the AKI patients without other serious complications, some will regularly receive blood purification therapy, which may be helpful to clear urea nitrogen and SCr effectively [8, 9]. Furthermore, this study also showed different degree of electrolyte disturbance on admission, such as hypokalemia and hyponatremia, while their levels were not correlated with prognosis.

Previous studies have demonstrated that the liver function injury can be appeared in the patients with HFRS [10–12]. This study also showed that most HFRS patients manifested abnormal levels of ALT and AST on admission. High level of AST and ALT were correlated with prognosis, and the level of AST was also higher than that of ALT, which may be related to the location of AST. In addition to the release by injury liver cell, multiple organ injuries such as myocardium, muscle, kidney and pancreas can also cause the release of AST. Among them, the myocardial cell injury has a greater impact on the elevation of AST, and the viral myocarditis and heart failure caused by Hantavirus can reflect the severity of the patient's disease and may be directly related to the prognosis [13]. This study also showed that hyperbilirubinemia was more common in the death group, suggesting that severe HFRS patients may have obvious decrease in the liver metabolic function. Furthermore, most of the coagulation factors can be synthesized in the liver, and their half-life is short and degradation is fast, so the liver injury can further aggravate coagulation and fibrinolysis dysfunction.

Overexpression of inflammatory cytokines is usually involved in the pathophysiological process of the HFRS patients [2, 4]. The increased expression and imbalance of cytokines and chemokines further aggravate the vascular endothelial injury, which in turn leads to increased permeability [4]. Our results showed that the level of WBC increased during the acute stage. The increasing of WBC was correlated with the prognosis and the level of WBC in the dead cases can reach to more than 30 × 10^9^/L, suggesting that the early severe inflammatory cytokines storm might play an important role in the pathogenesis of HFRS.

Coagulation disorders are one of the main pathophysiological changes in HFRS, with hemorrhage as common clinical manifestation. The mechanism of bleeding in HFRS has not been fully elucidated. Studies have shown that coagulation and inflammation are mutually causal, and together as part of the innate immune response [14], which can be activated during viral infection. Inflammation itself can also promote thrombosis through various mechanisms, finally enabling the unlimited formation of microvascular thrombus, and ultimately cause DIC and multiple organ failure [15]. A study of PUUV infection showed that 28% of the HFRS patients developed DIC during the early stage of the disease [16]. Another study on HCPS patients showed that DIC was also an important clinical manifestation [17]. In this study, we used ISTH DIC unified scoring system to analyze and found that 27.30% of the HFRS patients enrolled manifested DIC on admission, which was correlated with the prognosis (death), and was similar with previous study [18]. This result indicates that extensive coagulation activation can occur early in the course of HFRS, demonstrating rapid disease progress and is closely related to the prognosis, which requiring early intervention.

Thrombocytopenia is also a major factor in the disorder of coagulation mechanism of HFRS. Current studies suggest that decreased PLT is one of the factors involved in the vascular endothelial injury and increased permeability [19]. A reduction in PLT on admission had been shown to help predict disease severity in HCPS patients [20]. This study showed that most HFRS patients have significant decrease of PLT early in the course of the disease, and severe PLT reduction was more common in the dead cases. Until now, the mechanism of PLT reduction in HFRS is still unclear. Some studies suggested that the reduction of PLT was related to its continuous consumption [21]; Others indicated that the reduction of PLT was mainly related to adhesion to the surface of endothelial cells infected with hantavirus [22].

This study showed that PT and APTT can reach the maximum on the first day after admission. Prolongation of PT and APTT reflects the activation of coagulation factors, and prolonged PT can be considered as a predictor of death, suggesting that coagulation activation was more prominent in the dead patients, which was consistent with the study in HCPS [23]. The continuous activation and consumption of coagulation factors and PLT may indicate an increase in the body's production of thrombin. Thrombin is a "double-edged sword", an appropriate amount can promote blood coagulation, and an excessive amount may aggravate the activation of the inflammatory response, thereby increasing the permeability of blood vessels [24]. PLT activation and blood coagulation processes corelated each other [25]. Previous studies have found that increased thrombin generation in the patients with PUUV infection and SNV infection can predict the severity of the disease, and the increase in thrombin was more obviously in critically ill patients [26, 27].

As an acute phase protein mainly produced by liver, Fib is a bridge for platelets aggregation and a target of thrombin, playing an important role throughout blood coagulation. This study showed that the reduction of Fib was closely related to the disease severity, which was consistent with the results of some previous studies [28], while there were also studies with different result [16]. In a clinical study of the patients with PUUV infection, the median level of Fib was 4.2 g/L [16], which was much higher than the result in our study. The reason may be related to the different viral serotypes. The majority of the cases enrolled in this study were HTNV-infected patients, whose viral pathogenicity was significantly higher than that of the patients infected by PUUV, and the degree of coagulation and fibrinolysis activation caused by HTNV infection may be stronger. In addition to persistent consumption of Fib, the decreased capacity of liver synthesis will also cause further reduction of Fib. Fib was strongly associated with poor prognosis in HFRS patients. Therefore, clinical attention should be paid to the dynamic monitoring and evaluation of Fib.

In addition to coagulation activation, fibrinolytic activity is also activated in the HFRS patients. The fibrinolytic process is a series of protease-catalyzed reactions, and the final product is plasmin. Plasmin can degrade microthrombi generated by coagulation activation and prevent intravascular fibrin deposition, while uncontrolled plasmin production can also cause systemic hemorrhage within minutes [29, 30]. Fibrinogen degradation products and fibrin degradation products are as all referred to as FDP, and D-dimer is the specific degradation products of the cross-linked fibrin. Previous studies have shown that HFRS patients with HTNV infection had low expression of plasminogen and high expression of FDP [8, 10]. This study also showed that the levels of D-dimer and FDP were increased on admission, while there was no significant correlation with the prognosis. This result was consistent with previous studies on other hantavirus infections (PUUV and SNV) [31, 32].

Different hantavirus serotypes may cause different degrees of fibrinolytic activation in the body. Studies have shown that the median D-dimer in the patients with PUUV infection is 4.8 mg/L [16], and 1.82 mg/L in the HCPS patients [31]. The possible reason is that if a large number of thrombus forms in the body but does not dissolve, the increase in D-dimer may be mild. It can be seen that low fibrinolysis may be more common in the patients with HCPS, which was associated with less severe bleeding complications in the HCPS patients, while hypercoagulability and severe organ dysfunction were the more common manifestations in HCPS [33]. In contrast to HCPS, hyperfibrinolysis was obvious in the patients with PUUV infection. Some studies have shown that increased fibrinolysis activation in the patients with PUUV infection during the acute stage, and excessive fibrinolysis in PUUV patients can reduce the formation of microthrombi, and have a preventive effect on organ dysfunction and may be benefit to clinical recovery [16], Another study has shown that increased fibrinolysis was associated with the increased bleeding risk in the patients with PUUV infection [34]. Other studies also shown that patients with high fibrinolytic activation are prone to have bleeding complications, but the incidence of organ dysfunction is low, and patients with low fibrinolytic activation are more prone to have organ dysfunction [35]. These results may largely explain the enhanced bleeding risk observed in some patients with HFRS.

Compared with PUUV infection, patients with HTNV infection have more serious bleeding complications. This study also showed that HTNV-infected HFRS patients have hyperfibrinolysis during the early stage. First, on the 7th day after admission, the levels of PLT, PT, APTT and Fib gradually became normal, while the levels of D-dimer and FDP remained high. In addition, the proportion of D-dimer and FDP abnormalities are higher than PT, APTT, Fib, and more dramatic range of increase. This result may at least partially indicate that fibrinolysis activation was greater than coagulation activation in the patients with HTNV infection during the course of disease progression, tilting the balance towards fibrinolysis. However, in this study, hyperfibrinolysis is not associate with disease prognosis, its relationship with bleeding complications needs further study. Finally, we ventured to speculate that HTNV could directly activate fibrinolysis independently of the coagulation system and it could be a common manifestation of HTNV infection. The problem which need to be emphasized was that this study was retrospective, and some conclusions might be still speculative. The mechanism of hyperfibrinolysis caused by HTNV infection has not been elucidated, and further exploration and research are needed in the future.

This study has some limitations. First, the number of cases enrolled is still relatively small. In recent years, with the continuous updating and development of intensive care and treatment technology, the incidence of HFRS in Xi'an has decreased gradually. Second, some HFRS patients enrolled in this study may have received different treatment prior to admission because of different timing for seeking medical assistance, some laboratory parameters may change rapidly during the disease progression, so comparing their expression at different timing may lead to based error. Third, some parameters (APTT, TT) are limited by their upper limit of detection and may underestimate the differences between the two groups. In addition, this study is a retrospective design and has no validation analysis. Therefore, it is necessary to conduct multicenter, prospective cohort studies with larger and follow-up samples in the future.

## Conclusions

Detection of PT, Fib and TBIL on admission is necessary, which might be benefit to early predicting prognosis. It is also important to pay attention to the dynamic coagulation disorders and hyperfibrinolysis during the early stage in the severe HFRS patients.

## Data Availability

The datasets used and/or analyzed during the current study are available from the corresponding author on reasonable request.

## Abbreviations

HFRS: Hemorrhagic fever with renal syndrome
AKI: Acute kidney injury
MODS: Multiple organ dysfunction syndrome
ROC curve: Receiver operating characteristic curve
AUC: Area under the ROC curve
WBC: White blood cells
PLT: Platelet
HCT: Hematocrit
ALB: Albumin
ALT: Alanine aminotransferase
AST: Aspartate aminotransferase
PT: Prothrombin time
APTT: Activated partial thromboplastin time
Fib: Fibrinogen
SCr: Serum creatinine
DIC: Disseminated intravascular coagulation
HCPS: Hantavirus Cardiopulmonary Syndrome
SNV: Sin Nombre virus
HTNV: Hantaan virus
SEOV: Seoul virus
PUUV: Puumala virus
NE: Epidemic nephropathy
ISTH: International Society of Thrombosis and Hemostasis
VIF: Variance inflation factor
CI: Confidence interval
CRP: C-reactive protein
TT: Thrombin time
FDP: Fibrinogen degradation products
TBIL: Total bilirubin
